# Decreased Circulating Gonadotropin-Releasing Hormone Associated with Keratoconus

**DOI:** 10.3390/cells13201704

**Published:** 2024-10-15

**Authors:** Paulina Escandon, Alexander J. Choi, Steve Mabry, Sarah E. Nicholas, Rebecca L. Cunningham, Liam Redden, David A. Murphy, Kamran M. Riaz, Tina B. McKay, Dimitrios Karamichos

**Affiliations:** 1 North Texas Eye Research Institute, University of North Texas Health Science Center, 3430 Camp Bowie Blvd, Fort Worth, TX 76107, USA; pescandonb@gmail.com (P.E.); alexander.choi@unthsc.edu (A.J.C.); steve.mabry@unthsc.edu (S.M.); sarah.nicholas@unthsc.edu (S.E.N.); 2 Department of Pharmaceutical Sciences, University of North Texas Health Science Center, 3500 Camp Bowie Blvd, Fort Worth, TX 76107, USA; rebecca.cunningham@unthsc.edu; 3 Department of Ophthalmology, Dean McGee Eye Institute, University of Oklahoma Health Sciences Center, Oklahoma City, OK 73104, USA; lm766098@dal.ca (L.R.); dave.am999@gmail.com (D.A.M.); kamran-riaz@dmei.org (K.M.R.); 4 Department of Anesthesia, Critical Care and Pain Medicine, Massachusetts General Hospital and Harvard Medical School, Boston, MA 02116, USA; 5 Department of Pharmacology and Neuroscience, University of North Texas Health Science Center, 3500 Camp Bowie Blvd, Fort Worth, TX 76107, USA

**Keywords:** keratoconus, crosslinking, estrogen, corneal thinning, saliva, biomarkers

## Abstract

Keratoconus (KC) is a corneal thinning dystrophy that leads to visual impairment. While the cause of KC remains poorly understood, changes in sex hormone levels have been correlated with KC development. This study investigated circulating gonadotropin-releasing hormone (GnRH) in control and KC subjects to determine if this master hormone regulator is linked to the KC pathology. Plasma and saliva were collected from KC subjects (n = 227 and n = 274, respectively) and non-KC controls (n = 58 and n = 101, respectively), in concert with patient demographics and clinical features. GnRH levels in both plasma and saliva were significantly lower in KC subjects compared to controls. This finding was retained in plasma when subjects were stratified based on age, sex, and KC severity. Control and KC corneal fibroblasts (HKCs) stimulated with recombinant GnRH protein in vitro revealed significantly increased luteinizing hormone receptor by HKCs and reduced expression of α-smooth muscle actin with treatment suggesting that GnRH may modulate hormonal and fibrotic responses in the KC corneal stroma. Further studies are needed to reveal the role of the hypothalamic–pituitary–gonadal axis in the onset and progression of KC and to explore this pathway as a novel therapeutic target.

## 1. Introduction

Keratoconus (KC) is a disorder characterized by progressive protrusion and thinning of the cornea, leading to impaired vision. KC typically manifests during puberty, with a remarkably unpredictable rate of progression and disease severity, extending 10–20 years [1,2,3,4,5]. The specific underlying factors for KC onset are not well understood; however, historically, it is considered multifactorial with genetic and environmental components [6,7,8,9,10]. Genetic factors associated with KC include familial inheritance [11], genetic disorders [12,13], and being a twin [14,15,16]. Environmental factors associated with KC include contact lens wear [17] and chronic eye rubbing [18,19,20]. KC has also been linked to numerous systemic diseases, such as Down’s syndrome [21,22,23], hypothyroidism [24], and sleep apnea [25,26]. In recent years, hormone-related imbalances have been associated with the onset and progression of KC [27,28,29,30]. Pregnancy-induced onset or progression of KC is believed to be triggered in women predisposed with inherently thinner corneas in concert with hormonal changes that occur during pregnancy [31,32,33].

The epidemiological associations between KC and hormone-related conditions have been supported by molecular differences observed in KC subjects. Altered androgen and estrogen hormone receptors expression have been reported in the KC corneal epithelium [34,35]. Our group has reported significant downregulation of salivary and plasma estriol and estrogen and elevated dehydroepiandrosterone sulfate in KC subjects [27,36]. Interestingly, decreased levels of a hormonally-regulated protein known as prolactin-induced protein, has also been associated with KC [37,38]. Differences in the gonadotropins luteinizing hormone (LH) and follicle-stimulating hormone (FSH) ratio have been associated with KC [39,40]. In vitro, we have observed significant changes in KC-derived corneal stromal cell proliferation, expression of sex hormone receptors, fibrosis, proteoglycans, and members of the gonadotropin signaling pathway following stimulation by exogenous LH, when compared to control-derived corneal stromal cells [39,40]. Interestingly, exogenous FSH did not elicit differential responses [39,40]. Further investigations into regulatory pathways upstream of LH and FSH are needed to determine the cellular and molecular origins of KC and the reported associations with hormonal imbalance.

Gonadotropin-releasing hormone (GnRH) is a vital hormone for reproductive health that is synthesized and released by neurons within the hypothalamus [41,42]. GnRH is responsible for stimulating the pituitary gland to secrete the gonadotropins LH and FSH, regulating both endocrine function and gamete maturation in the gonads [43,44,45,46]. GnRH secretion is functional after birth; however, GnRH levels are low in children and rise during puberty [47,48,49,50]. During the onset of puberty, sex hormones, such as testosterone, estrogen, and progesterone, function to control GnRH levels [49,50]. GnRH is under negative feedback control [51]. For example, when sex hormones are high, the body secretes less GnRH; however, when sex hormones are low, GnRH production and secretion increases. In turn, increasing levels of GnRH stimulate the production of LH and FSH, which results in sex gland maturity, fertility, and function [44,45,46,50].

This study investigated circulating levels of GnRH, as a master hormone regulator upstream of LH and FSH production, in control and KC subjects, to determine associations between differential levels and the KC pathology. Further in vitro studies were performed to reveal the effects of exogenous GnRH on corneal fibroblasts derived from non-KC controls (HCFs) and KC subjects (HKCs). This paper provides an insight into a role for circulating systemic factors and the hypothalamic–pituitary–gonadal axis in the pathophysiology underlying KC.

## 2. Materials and Methods

### 2.1. Plasma and Saliva Collection

Plasma collection: Donors provided blood samples collected in 10 mL EDTA-coated tubes (BD vacutainer, Franklin Lakes, NJ, USA). The tubes were then inverted to ensure mixture of the whole blood sample and centrifuged at 1300× *g* for 10 min at 4 °C to separate the plasma layer [36]. The plasma samples were then transferred into sterile microcentrifuge tubes and stored at −80 °C prior to processing. Saliva collection: Donors rinsed their mouths with water then collected saliva via passive drool in 15 mL tubes (VWR, Radnor, PA, USA), as previously described [27,38,52]. The saliva samples were then aliquoted and stored at −80 °C prior to processing.

### 2.2. Enzyme-Linked Immunosorbent Assay (ELISA)

GnRH levels were examined in plasma and saliva samples according to the manufacturer’s instructions using commercially available enzyme-linked immunosorbent assay (ELISA) kits, such as the Human Gonadotropin-releasing Hormone ELISA Kit-Cat. # MBS161281 (MyBioSource, San Diego, CA, USA) and the Human GnRH ELISA Kit-Cat. # MBS2515500 (MyBioSource, San Diego, CA, USA).

### 2.3. Plasma ELISA

In brief, 50 µL of prepared standards and 40 µL of plasma sample were run in duplicate in the pre-coated 96-well plate. Following this, 10 µL of anti-GnRH antibody were added to each sample well; note that no antibodies were added to the standard wells. A total of 50 µL of streptavidin–HRP was then added to all sample and standard wells, mixed, covered with a plate sealer, and incubated for 60 min at 37 °C. Following incubation, the ELISA plate was rinsed five times by adding 350 µL of washing buffer using a Stat-Matic plate washer (ThermoFisher Scientific, Waltham, MA, USA) into each well, soaked for 1 min, removed, and pat dry plate with clean absorbent paper towels. Following the rinsing step, 50 µL of substrate solutions A and B were added to each well; the plate was then covered in foil to protect it from light and incubated for 10 min at 37 °C. Lastly, 50 µL of a stop solution was added to each well, gently mixed, and read immediately at 450 nm using a BioTek EPOCH2 microplate reader (BioTek, Winooski, VT, USA). BioTek Gen 5 software, version 3.10 was programmed to calculate each sample’s results by plotting the optical density values using a four-parameter logistic curve fit.

### 2.4. Saliva ELISA

In brief, 50 µL of prepared standards and 50 µL of each saliva sample were run in duplicate in the 96-wells plate. Immediately after, 50 µL of a biotinylated detecting antibody solution was added to all wells. The ELISA plate was then inoculated with a sealer for 45 min at 37 °C. Next, the solution was discarded from each well and pat dry against clean absorbent paper towels. Following incubation, the ELISA plate was rinsed three times by adding 350 µL of washing buffer using a Stat-Matic plate washer (ThermoFisher Scientific, Waltham, MA, USA) into each well, soaked for 1 min, removed, and pat dry plate with clean absorbent paper towels. The ELISA plate was then incubated with 100 µL of horseradish peroxidase (HRP) conjugated working solution in each well, covered with a new plate sealer, and incubated for 30 min at 37 °C. After incubation, the solution was discarded, and the rinsing process described above was conducted five times. Following the rinsing step, 90 µL of substrate reagent was added to each well; the plate was then covered in foil to protect from light and incubated for 15 min at 37 °C. Finally, 50 µL of a stop solution was gently mixed into each well. As described above, the plate’s optical density was read immediately with a BioTek EPOCH2 microplate reader (BioTek, Winooski, VT, USA).

### 2.5. Isolation of Corneal Stromal Fibroblasts

Healthy corneal stromal fibroblasts (HCFs) were isolated from healthy cornea donors, as previously described [53]. The keratoconus corneal stromal cells (HKCs) were isolated from KC cornea donors. Briefly, corneas were scraped with a razor to remove the cornea’s epithelial and endothelial layer, followed by rinsing with sterile phosphate buffer saline (PBS; ThermoFisher Scientific, Waltham, MA, USA). The cornea’s stromal layer was cut into 2 × 2 mm pieces and spaced evenly into a T-25 flask, followed by incubation for 45 min at 37 °C in 5% CO_2_. Following adhesion, complete media (CM), namely Eagle’s Minimum essential media (EMEM) (American Type Culture Collection, Manassas, VA, USA) supplemented with 10% fetal bovine serum (FBS) (Atlanta Biologicals, Flowery Branch, GA, USA) and 1% antibiotic/antimycotic (AA) (Life Technologies, Gran Island, NY, USA), was added to the T-25 flask for one week. The media were changed every two days after initial adhesion of the cornea’s stromal until cells growth reached ~80% confluency, following isolation for further expansion/processing of the cells. For this study, only cell passages between 3–6 were used.

### 2.6. Cell Culture and Exogenous Stimuli

HCFs and HKCs were cultured in a T-175 flask with CM until ~90% confluency. Cells were passaged using 0.05% trypsin–EDTA, before cell counting with Countless II (Invitrogen, ThermoFisher Scientific, Waltham, MA, USA) and seeding for 2D and 3D cell culture study.

### 2.7. 2D Cell Culture

2D HCFs were plated at a 0.5 × 10^6^ cells/well seeding density in 1 mL of CM on a clear flat-bottom 6-well plate at 37 °C in 5% CO_2_ overnight. HCFs (n = 3) were then stimulated with recombinant human GnRH protein (rGnRH; ab112295; Abcam, Cambridge, MA, USA) concentrations (2 ng/mL, 10 ng/mL, 25 ng/mL, 50 ng/mL, 100 ng/mL, and 500 ng/mL) for 48 h; protein concentrations were decided based on human GnRH levels during the human lifespan [54,55,56,57,58,59]. HCFs with CM-only served as controls. After 48 post-stimulation, the protein was extracted from the cell cultures, and analysis was performed to detect the GnRH receptor (GnRHR; rabbit polyclonal, ab183079, Abcam Cambridge, MA, USA).

A second study was executed where HCFs and HKCs were plated at a 0.5 × 10^6^ cells/well seeding density in 1 mL of CM on a clear flat-bottom 6-well plate at 37 °C in 5% CO_2_ overnight. Following cell adhesion, the cultures (n = 3 per cell type) were stimulated with recombinant human GnRH protein (rGnRH) (ab112295; Abcam, Cambridge, MA, USA) concentrations (1 ng/mL, 2 ng/mL, 4 ng/mL, 6 ng/mL, 8 ng/mL, and 10 ng/mL) for 48 h. Protein concentrations were selected based on the GnRHR protein expression analysis from the first set of rGnRH stimulation series on HCFs. Cultures with CM-only for each cell type served as controls. After 48 h of stimulation, cell culture protein was extracted, and analysis of GnRHR (rabbit polyclonal, ab183079, Abcam Cambridge, MA, USA) was used to determine the optimal rGnRH concentrations for further testing using 3D cultures. Discussion on the rationale of the chosen rGnRH concentrations used in this study is mentioned below in Section 3 and Section 4.

### 2.8. 3D Cell Culture

HCFs and HKCs were seeded in clear flat-bottom 6-well plates (ThermoFisher Scientific, Waltham, MA, USA) at a concentration of 1 × 10^6^ per well, and grew confluent over 24 h incubation at 37 °C with 5% CO_2_ with CM. Cultures (n = 4) were then stimulated with three concentrations (1 ng/mL, 4 ng/mL, and 8 ng/mL) of rGnRH protein (ab112295; Abcam, Cambridge, MA, USA), in Vitamin C media (VCM), i.e., EMEM supplemented with 10% FBS, 1% AA, and 0.5 mM stable vitamin C (0.5 mM 2-O-α-D-glucopyranosyl-L-ascorbic acid, Sigma-Aldrich, St. Louis, MO, USA). Cultures with VCM alone were considered controls. Fresh VCM and/or rGnRH was provided to the cultures every 48 h for four weeks, followed by protein extraction and further processing.

### 2.9. Protein Extraction and Quantification

Protein from 2D and 3D cell cultures was extracted and lysed using 1× radioimmunoprecipitation buffer assay and protease inhibitor (RIPA-PI, Sigma-Aldrich, St. Louis, MO, USA). In brief, 100 µL of RIPA-PI were added to cultures after being washed with PBS (ThermoFisher Scientific, Waltham, MA, USA) twice. Samples were then incubated for 30 min at 4 °C in an ice bucket. Lysates were centrifugated for 15 min at 12,000 RPM at 4 °C, and the supernatant was collected for protein quantification.

Proteins were quantified using a Pierce™ BCA Protein Assay Kit and bovine serum albumin standards (ThermoFisher Scientific, Waltham, MA, USA). In brief, 10 µL of standards and samples were added to a Corning™ Costar™ 96-well plate (ThermoFisher Scientific, Waltham, MA, USA), followed by 200 µL of Pierce™ BCA reagent A and B solution to each well, and then incubated for 30 min at 37 °C with 5%CO_2_. The BioTek EPOCH2 microplate reader (BioTek, Winooski, VT, USA) was used to measure the samples and standards absorbances values. Data were plotted by BioTek Gen5 Software, version 3.10, and protein concentrations were determined for western blot analysis.

### 2.10. Western Blot

Proteins were analyzed by SDS-PAGE at 225 volts for 30 min in Novex 4–20% Tris-glycine Mini Wedge 12-well gels (Invitrogen, ThermoFisher Scientific, Waltham, MA, USA). Gels were transferred to nitrocellulose membranes (NCMs) using iBlot 2 Dry Blotting System (Invitrogen, ThermoFisher Scientific, Waltham, MA, USA). NCMs were then blocked with1 X Blocker™ FL fluorescent blocking buffer (ThermoFisher Scientific, Waltham, MA, USA) for 1 h at room temperature on a rocker, followed by primary antibody incubation overnight at 4 °C. In addition, all NCMs were stained with β-actin for housekeeping to normalize based on the protein expression. After washing with TBST three times for 5 min each, the secondary antibody was added and inoculated for 1 h. Detailed information of all primary/secondary antibodies are listed in Table 1. Finally, all NCMs were imaged with the iBright FL 15000 imaging system (ThermoFisher Scientific, Waltham, MA, USA). iBright analysis software, version 1.8.1 (ThermoFisher Scientific, Waltham, MA, USA) was used to analyze images, and the results were normalized to the expression of β-actin housekeeping and plotted.

### 2.11. Immunofluorescence Microscopy

HCFs and HKCs were plated at a 0.1 × 10^6^ cells/well seeding density in 200 μL of CM on a glass 12-well chamber slide at 37 °C in 5% CO_2_ overnight. Following overnight cell adhesion, the cultures (n = 4 per cell type) were stimulated with three rGnRH concentrations: 1 ng/mL, 4 ng/mL, and 8 ng/mL for 48 h. CM-only was added to all control wells. After 48 h, cells were fixed with 200 μL 4% paraformaldehyde (PFA) (AA433689L, ThermoFisher Scientific, Waltham, MA, USA) in PBS (14-200-075, ThermoFisher Scientific, Waltham, MA, USA) for 30 min at room temperature. The PFA solution was discarded from each well and patted dry against clean absorbent paper towels. To permeabilize cells, 5% TritonX-100 in PBS was added to each well for 30 min at room temperature. The 5% TritonX-100 (AAA16046AE, ThermoFisher Scientific, Waltham, MA, USA) solution was discarded by inverting over the sink and tapping against clean absorbent paper towels, followed by blocking with 3% Milk (NC9121673, ThermoFisher Scientific, Waltham, MA, USA) in PBS for 1 h at room temperature. Staining used GnRHR rabbit polyclonal antibody (ab183079, Abcam, Cambridge, MA, USA) and Alexa568 anti-rabbit IgG (ab175470, Abcam, Cambridge, MA, USA) by inoculating for 1 h at room temperature. Finally, the slides were covered with mounting media containing DAPI stain (ab104139, Abcam, Cambridge, MA, USA) and kept in the dark at 4 °C until imaged using a Keyence BZ-X700 series microscope (Keyence, Itasca, IL, USA). Images were analyzed using ImageJ (U. S. National Institutes of Health, Bethesda, MD, USA). Immunofluorescence signal intensity for GnRHR expression was determined with ImageJ by correcting the total fluorescence detected by subtracting out background signal, followed by comparisons of measured fluorescence intensity between cells.

### 2.12. Statistical Analysis

Experimental replicas reported as mean ± standard error of the mean or median with interquartile ranges based on distribution patterns and plotted with GraphPad Prism 9 (Graph Pad Software version 9.4.1., San Diego, CA). As all biological fluids data did not have Gaussian distribution, significance between two groups was determined using a Mann–Whitney test, and three or more groups were assessed by a Kruskal–Wallis test with Dunn’s multiple comparisons test. All other data were assessed by either unpaired *t*-test with Welch’s correction (two groups), or one-way analysis of variance (ANOVA) with Šídák’s multiple comparisons test (three or more groups). The analysis was noted to be significant when *p* < 0.05.

### 2.13. Study Approval

Research involving human subjects was approved by the North Texas Regional Institutional Review Board (Protocol #2020-031) and adhered to the Declaration of Helsinki. Healthy human corneas were obtained from the National Disease Research Interchange (NDRI, Philadelphia, PA). KC donor corneas were obtained from our clinical collaborator (KMR; Dean McGee Eye Institute, Oklahoma City, OK, USA). Saliva and plasma samples were collected from our clinical collaborator (KMR; Dean McGee Eye Institute, Oklahoma City, OK, USA). Written informed consent was obtained from all participants prior to collection. Donor information, including sex, age, ocular history, KC severity, and treatment, were collected for each sample by physicians.

## 3. Results

### 3.1. GnRH Plasma Analysis

**Figure 1:**
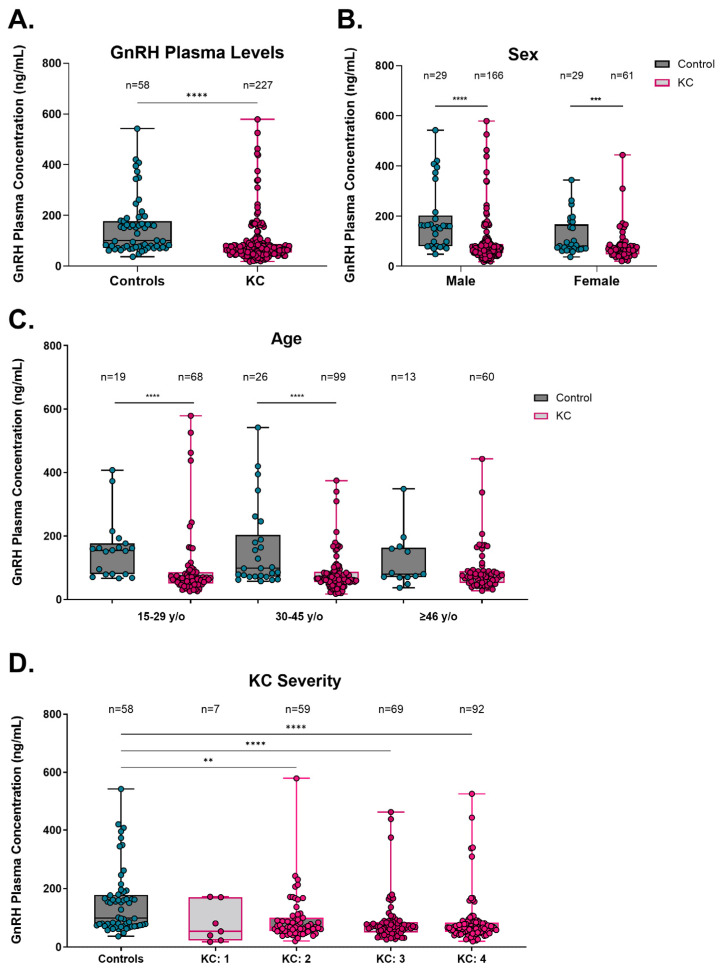
Plasma gonadotropin-releasing hormone (GnRH) in control and KC patients. (**A**) GnRH concentration in plasma for all control and KC patients tested. Sub-analyses of GnRH concentrations based on (**B**) sex, (**C**) age, and (**D**) KC severity. Data are shown in box-and-whisker plots, with the box represent the 25% quartile, median, and 75% quartile, and the line extending to the minimum and maximum values. Statistical significance evaluated by a non-parametric Mann–Whitney test (**A**–**C**). Statistical significance evaluated by a non-parametric Kruskal–Wallis test with Dunn’s multiple comparisons test (**D**). Significance indicated as ** *p* < 0.01, *** *p* < 0.001, and **** *p* < 0.0001.

To determine a baseline concentration of GnRH in the plasma of KC subjects, enzyme-linked immunoassays (ELISAs) were performed and compared to control patients without a diagnosis of KC (Figure 1). The overall GnRH plasma concentrations in KC subjects were significantly lower (median, interquartile range (IQR): 67.4 ng/mL, IQR 52.6–87.7 ng/mL) compared to control subjects (100.1 ng/mL, IQR 72.3–177.8 ng/mL, *p* < 0.0001, Figure 1A). Specifically, GnRH plasma concentration in both male and female KC subjects (67.3 ng/mL, IQR 53.4–89.8 ng/mL and 67.8 ng/mL, IQR 47.4–85.4 ng/mL, respectively) had lower GnRH plasma concentrations than sex-matched control subjects (159.9 ng/mL, IQR 80.0–202.0 ng/mL and 80.3 ng/mL, IQR 68.8–166.4 ng/mL, respectively, male *p* < 0.0001, female *p* = 0.0064, Figure 1B). Based on differential age distributions, KC subjects who were 15–29 years old (y/o) and 30–45 y/o had lower GnRH concentrations (67.3 ng/mL, IQR 50.8–86.2 ng/mL and 66.6 ng/mL, IQR 54.0–87.4 ng/mL, respectively) compared to age-matched controls (155.1 ng/mL, IQR 79.8–177.2 ng/mL and 98.3, IQR 72.3–203.3 ng/mL, respectively, 15–29 y/o *p* < 0.0001, 30–45 y/o *p* < 0.0001, Figure 1C).

To determine if the severity of KC might be associated with differential GnRH levels, KC subjects were stratified based on KC severity compared to controls. KC severities were identified by physicians based on the ABCD grading system, which integrates parameters based on anterior and posterior curvature 3 mm from the thinnest corneal pachymetry, thinnest corneal pachymetry and best corrected distance visual acuity [60]. Data showed that KC subjects with a severity score of 2 or greater had significantly lower GnRH concentrations in plasma when compared to control patients (*p* < 0.0001, Figure 1D). In addition, GnRH plasma concentration in KC subjects was significantly lower compared to control patients, independent of crosslinking (CXL) or corneal transplantation (*p* < 0.0001, Supplementary Figure S1). Finally, GnRH concentration in plasma was analyzed for KC subjects from samples collected before and after corneal crosslinking (CXL) treatment. KC subjects who had undergone CXL showed no significant differences in GnRH plasma concentration compared to controls, neither pre- nor post-CXL treatment (Supplementary Figure S2). Collectively, these results reveal significantly lower GnRH plasma levels in subjects with KC, independent of sex, KC severity, or treatment.

### 3.2. GnRH Saliva Analysis

**Figure 2:**
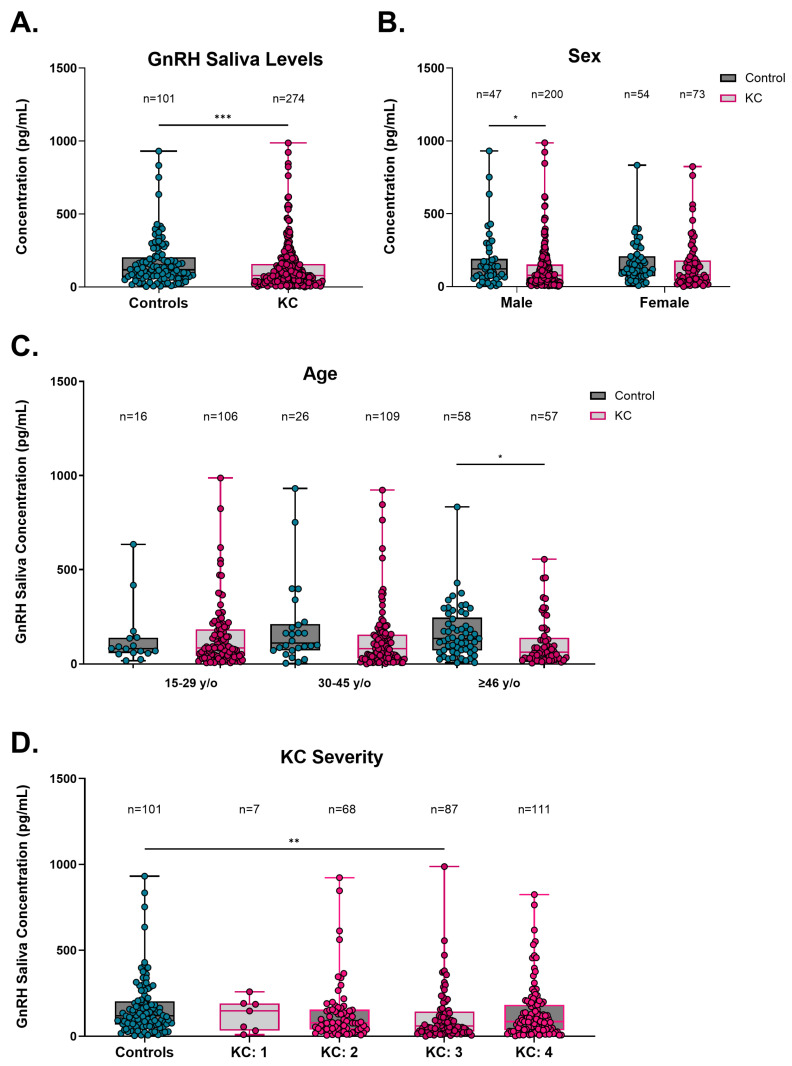
Saliva gonadotropin-releasing hormone (GnRH) in control and KC patients. (**A**) GnRH concentration in saliva for all control and KC patients tested. Sub-analyses of GnRH concentrations based on (**B**) sex, (**C**) age, and (**D**) KC severity. Data are shown in box-and-whisker plots, with the box representing the 25% quartile, median, and 75% quartile, and the line extending to the minimum and maximum values. Statistical significance evaluated by a non-parametric Mann–Whitney test (**A**–**C**). Statistical significance evaluated by a non-parametric Kruskal–Wallis test with Dunn’s multiple comparisons test (**D**). Significance indicated as * *p* < 0.05, ** *p* < 0.01, and *** *p* < 0.001.

To determine if the salivary GnRH levels agreed with those detected in plasma, ELISA was performed, comparing control and KC subjects (Figure 2). Consistent with GnRH concentrations in plasma, the overall GnRH saliva concentrations in KC subjects were significantly lower (77.7 pg/mL, IQR 32.3–158.0 pg/mL) compared to control subjects (118.6 pg/mL, IQR 67.7–203.6 pg/mL, *p* = 0.0007, Figure 2A). However, patients with both saliva and plasma samples tested had no significant difference observed, likely attributed to the smaller sample size (Supplementary Figure S3). GnRH saliva concentrations were lower in both male and female KC subjects (76.7 pg/mL, IQR 29.1–151.7 pg/mL and 80.7 pg/mL, IQR 38.2–179.2 pg/mL, respectively) compared to sex-matched control patients (121.1 pg/mL, IQR 61.0–190.4 pg/mL and 116.9 pg/mL, IQR 70.9–179.2 pg/mL, respectively), though this effect was only significant in the males (*p* = 0.0222, Figure 2B). When GnRH saliva concentrations were analyzed based on age, the results showed that KC subjects of ages greater than or equal to 46 y/o had significantly lower GnRH saliva concentration (61.2 pg/mL, IQR 28.2–138.5 ng/mL) when compared to age-matched control patients (133.6 pg/mL, IQR 70.7–244.9 pg/mL, *p* = 0.0040, Figure 2C) suggesting that older KC subjects may have a larger differential in baseline GnRH levels compared to subjects aged 15–45 y/o. GnRH concentration in the saliva of control patients was compared to KC subjects with different KC severities. KC subjects with a severity score of 3 had significantly lower GnRH concentrations in saliva when compared to control patients (*p* = 0.0084, Figure 2D).

Saliva GnRH concentration from KC subjects at other severity scores were not significantly lower than control patients. In addition, GnRH saliva concentrations in KC subjects were significantly lower compared to control patients, regardless of crosslinking (CXL) or corneal transplantation (*p* < 0.0001, Supplementary Figure S4). These results show that the GnRH plasma association with KC is retained in saliva, albeit with a smaller effect size and larger range than its detection in plasma.

### 3.3. GnRH Impact 2D Cell Culture Microenvironment

**Figure 3:**
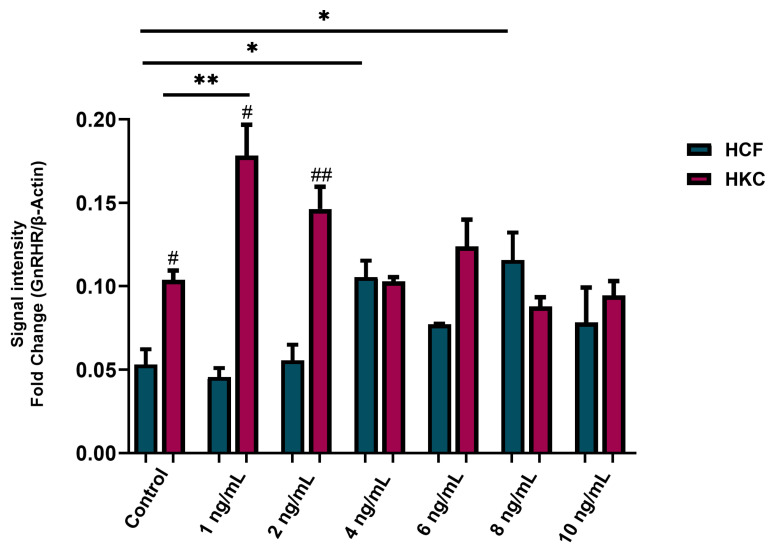
Protein expression of gonadotropin-releasing hormone receptor (GnRHR) in 2D HCFs and HKCs after being stimulated with 1 ng/mL, 2 ng/mL, 4 ng/mL, 6 ng/mL, 8 ng/mL, and 10 ng/mL rGnRH for 48 h. Data shown as mean ± standard error of the mean. n = 3 per group. Statistical significance of dose with HCFs and HKCs based on a one-way ANOVA with Šídák’s multiple comparisons test. Post hoc significance indicated as * *p* < 0.05 and ** *p* < 0.01. Statistical significance between HCFs and HKCs within dose determined by unpaired *t*-test with Welch’s correction. Significance indicated as # *p* < 0.05, ## *p* < 0.01.

Once we had shown that GnRH is affected systemically in subjects with KC, we began looking at its impact in the corneal microenvironment. To determine the best recombinant GnRH (rGNRH) concentrations for further testing, conventional 2D cultures of human corneal fibroblasts (HCFs) were utilized to evaluate the expression for GnRH receptor (GnRHR) as the determining factor for further experiments. The 48 h post-GnRH stimulation showed significant modulation of GnRHR protein expression when using 4 ng/mL and 8 ng/mL compared to the controls (Supplementary Figure S5). To narrow the range of optimal rGnRH concentrations, a similar study was executed utilizing the same 2D culture system, this time including both HCFs and HKCs. Data showed that GnRHR expression was significantly higher for HKCs stimulated with 1 ng/mL rGnRH (mean ± standard deviation = 0.18 ± 0.03 arbitrary units (a.u.)) compared to its own control (0.10 ± 0.01 a.u., *p* = 0.0007, Figure 3). Similarly, GnRHR protein expression was significantly higher for HCFs stimulated at 4 ng/mL (0.11 ± 0.02 a.u.) and 8 ng/mL (0.12 ± 0.03 a.u.) compared to its own control (0.05 ± 0.02 a.u., *p* = 0.0064, Figure 3). HKCs significantly elevated GnRHR protein expression compared to HCFs in controls (*p* = 0.0135), 1 ng/mL (*p* = 0.0135) and 2 ng/mL (*p* = 0.0071, Figure 3). No significant differences between HKCs and HCFs were observed at other doses of rGnRH.

**Figure 4:**
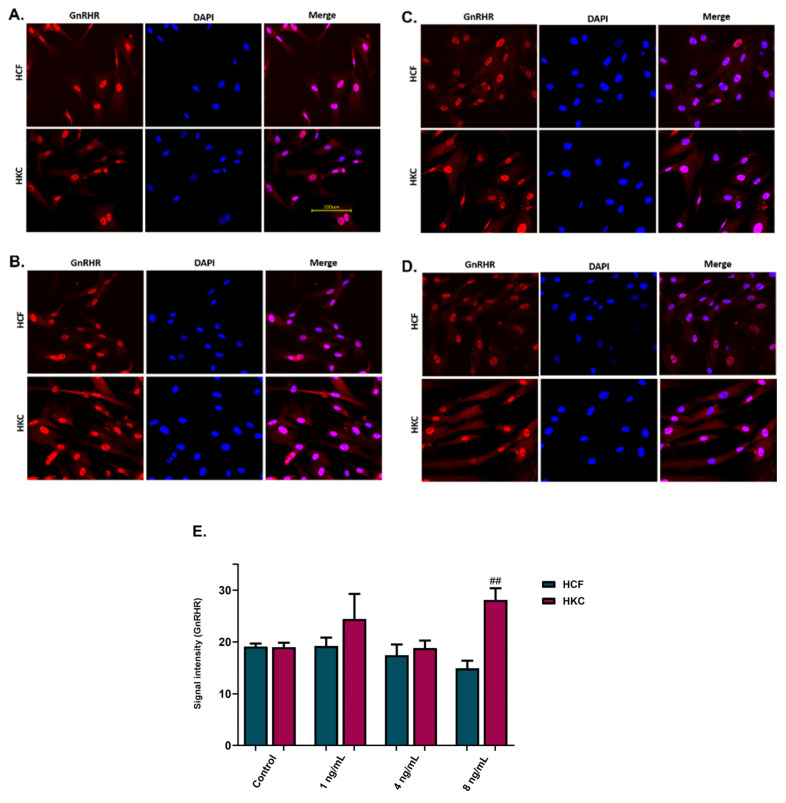
Immunofluorescence images and analysis for gonadotropin-releasing hormone receptor (GnRHR) expression in 2D GnRH stimulation of HCFs and HKCs. (**A**) The controls were not stimulated with GnRH; (**B**) 1 ng/mL; (**C**) 4 ng/mL; (**D**) 8 ng/mL; (**E**) immunofluorescence images’ signal intensity for GnRHR expression. Statistical significance between HCFs and HKCs within the dose determined by an unpaired *t*-test with Welch’s correction. Significance indicated as ## *p* < 0.01.

To visualize GnRHR expression in HCFs and HKCs, immunofluorescence staining was performed on the 2D cultures 48 h after stimulation with rGnRH. Stimulation with rGnRH was performed using the 1 ng/mL, 4 ng/mL, and 8 ng/mL concentrations (Figure 4A–D). Immunofluorescence signal intensity for GnRHR expression showed significantly higher GnRHR protein expression for HKCs stimulated with 8 ng/mL of GnRH compared to HCFs (*p* = 0.0043, Figure 4E). No significant differences were observed with HCF stimulated with GnRH at any of the concentrations tested, with the significant increase in GnRHR observed in HKCs suggesting that KC-derived cells are more responsive to GnRH stimulation compared to non-KC controls.

### 3.4. GnRH Impact in 3D Cell Culture Microenvironment

**Figure 5:**
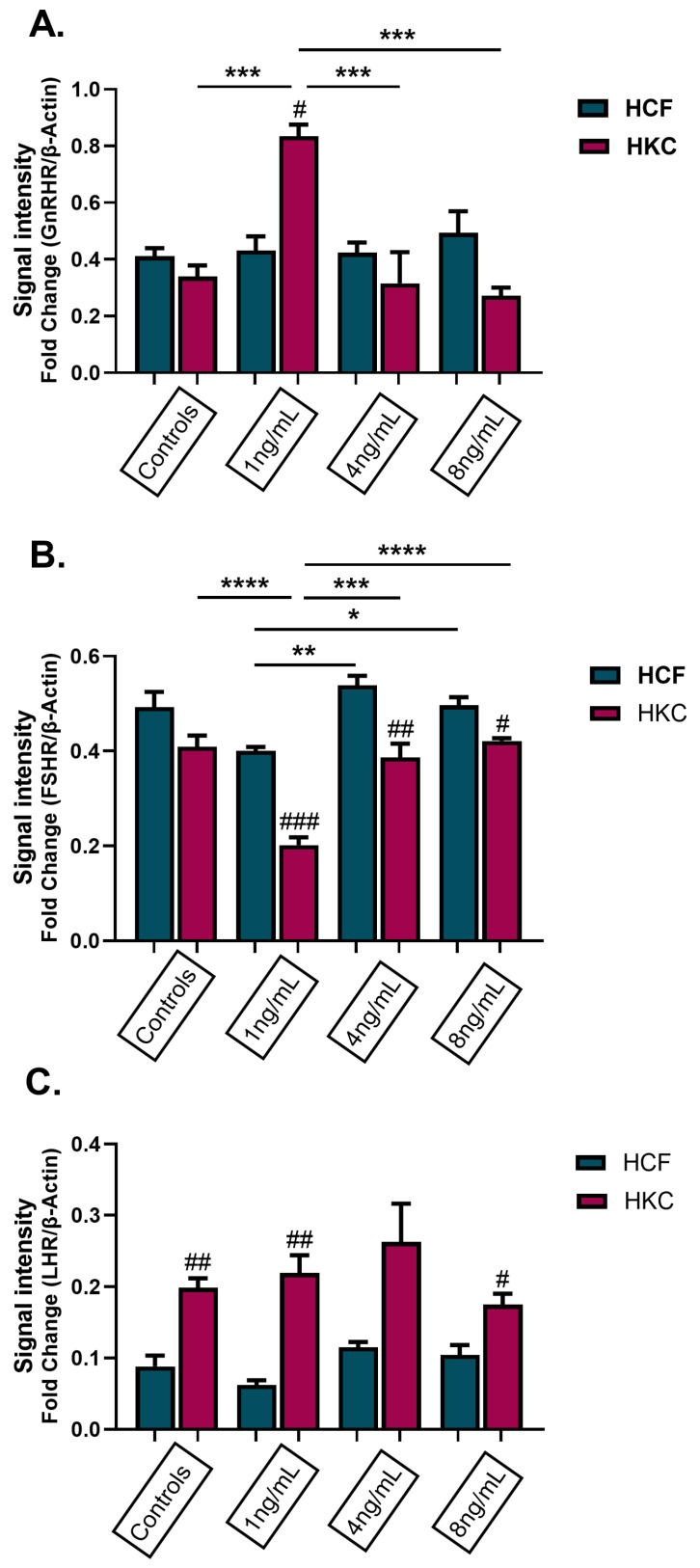
Protein expression of hormone receptors in 3D HCFs and HKCs following stimulation with 1 ng/mL, 4 ng/mL, and 8 ng/mL GnRH for 4 weeks. Protein expression of (**A**) GnRH receptor, (**B**) FSH receptor, and (**C**) LH receptor based on Western blot analysis. Statistical significance based on a one-way ANOVA with Šídák’s multiple comparisons test. Post hoc significance indicated as * *p* < 0.05, ** *p* < 0.01, *** *p* < 0.001, and **** *p* < 0.0001. Statistical significance between HCFs and HKCs within the dose determined by an unpaired *t*-test with Welch’s correction. Significance indicated as # *p* < 0.05, ## *p* < 0.01, and ### *p* < 0.001.

To study the effects of GnRH in a 3D in vitro corneal stromal model comparing HCFs and HKCs, protein expression of GnRHR, FSHR, and LHR were evaluated following stimulation with 1 ng/mL, 4 ng/mL, and 8 ng/mL rGnRH for 4 weeks (Figure 5). In HKCs stimulated with 1 ng/mL of rGnRH, there was a significant increase in GnRHR expression when compared to all other conditions (*p* = 0.0001, Figure 5A). GnRHR expression was also significantly greater in HKCs stimulated with 1 ng/mL compared to HCFs at the same dose (*p* = 0.0009, Figure 5A). FSHR expression results showed significantly higher expression in HCFs than HKCs when stimulated with 1 ng/mL (*p* = 0.0003), 4 ng/mL (*p* = 0.0067) and 8 ng/mL of rGnRH (*p* = 0.0151, Figure 5B). In addition, HCFs stimulated with 1 ng/mL of rGnRH were significantly lower than HCFs stimulated with 4 ng/mL and 8 ng/mL of rGnRH (*p* = 0.0042). Furthermore, HKCs stimulated with 1 ng/mL of rGnRH had significantly decreased FSHR expression when compared to control HKCs and HKCs stimulated with 4 ng/mL and 8 ng/mL of rGnRH (*p* < 0.0001, Figure 5B). Finally, LHR expression was significantly higher in HKC controls (*p* = 0.0016) and constructs stimulated with 1 ng/mL (*p* = 0.0061) and 8 ng/mL of rGnRH (*p* = 0.0138) when compared to HCFs with the same respective conditions (Figure 5C).

**Figure 6:**
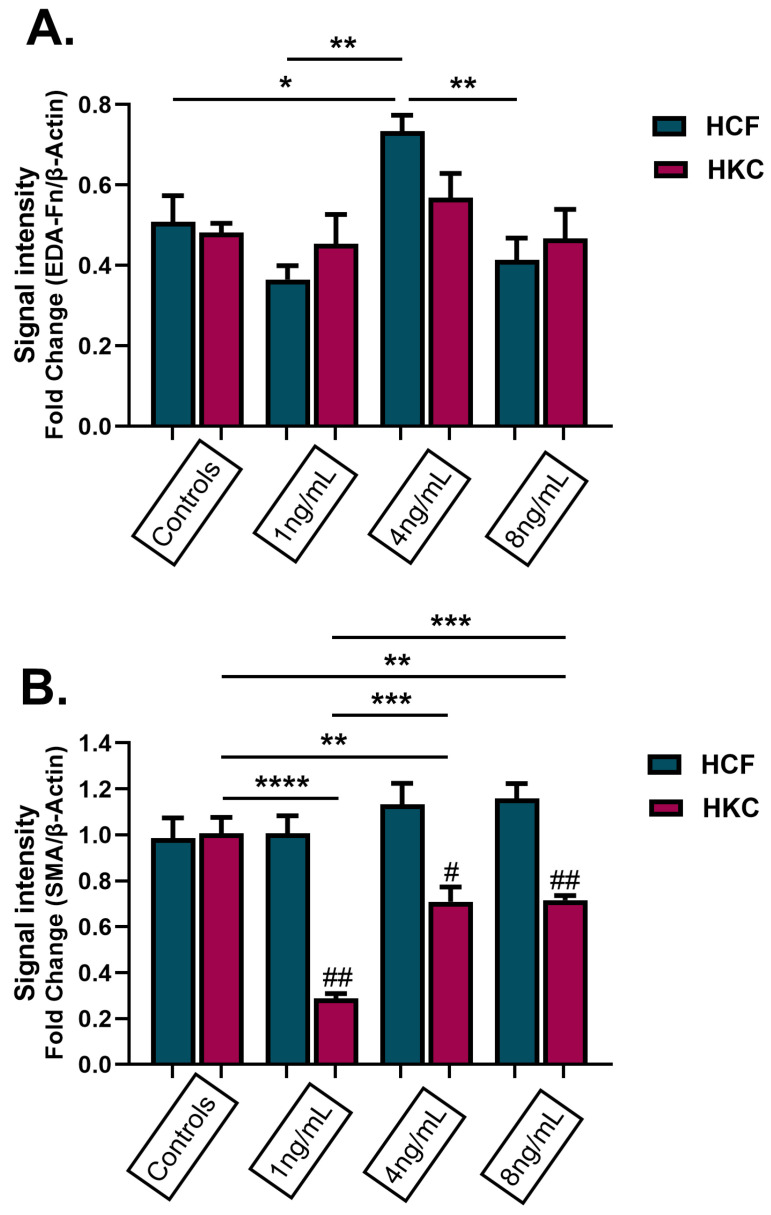
Protein expression of fibronectin and α-smooth muscle actin (SMA) in 3D HCFs and HKCs following stimulation with 1 ng/mL, 4 ng/mL, and 8 ng/mL GnRH for 4 weeks. (**A**) Results of EDA-Fn protein expression in HCFs and HKCs stimulated with GnRH. (**B**) Results of SMA protein expression in HCFs and HKCs stimulated with GnRH. Statistical significance based on a one-way ANOVA with Šídák’s multiple comparisons test. Post hoc significance indicated as * *p* < 0.05, ** *p* < 0.01, *** *p* < 0.001, **** *p* < 0.0001. Statistical significance between HCFs and HKCs within the dose determined by the unpaired *t*-test with Welch’s correction. Significance indicated as # *p* < 0.05, ## *p* < 0.01.

The results of the expression of fibrous proteins in HCFs and HKCs after being stimulated with 1 ng/mL, 4 ng/mL, and 8 ng/mL of rGnRH for 4 weeks are shown in Figure 6. The results showed that HCFs stimulated with 4 ng/mL of GnRH had a significantly increased expression of fibronectin (EDA-Fn) compared to control HCFs and all other doses of rGnRH (*p* = 0.0011, Figure 6A). In addition, α-smooth muscle actin (SMA) expression was significantly higher in HCFs stimulated with 1 ng/mL (*p* = 0.0015), 4 ng/mL (*p* = 0.0105), and 8 ng/mL (*p* = 0.0045) than in HKCs at the same doses (Figure 6B). For HKCs, expression of SMA was significantly higher in the untreated control, and lowest with 1 ng/mL of GnRH treatment (*p* < 0.0001, Figure 6B).

**Figure 7:**
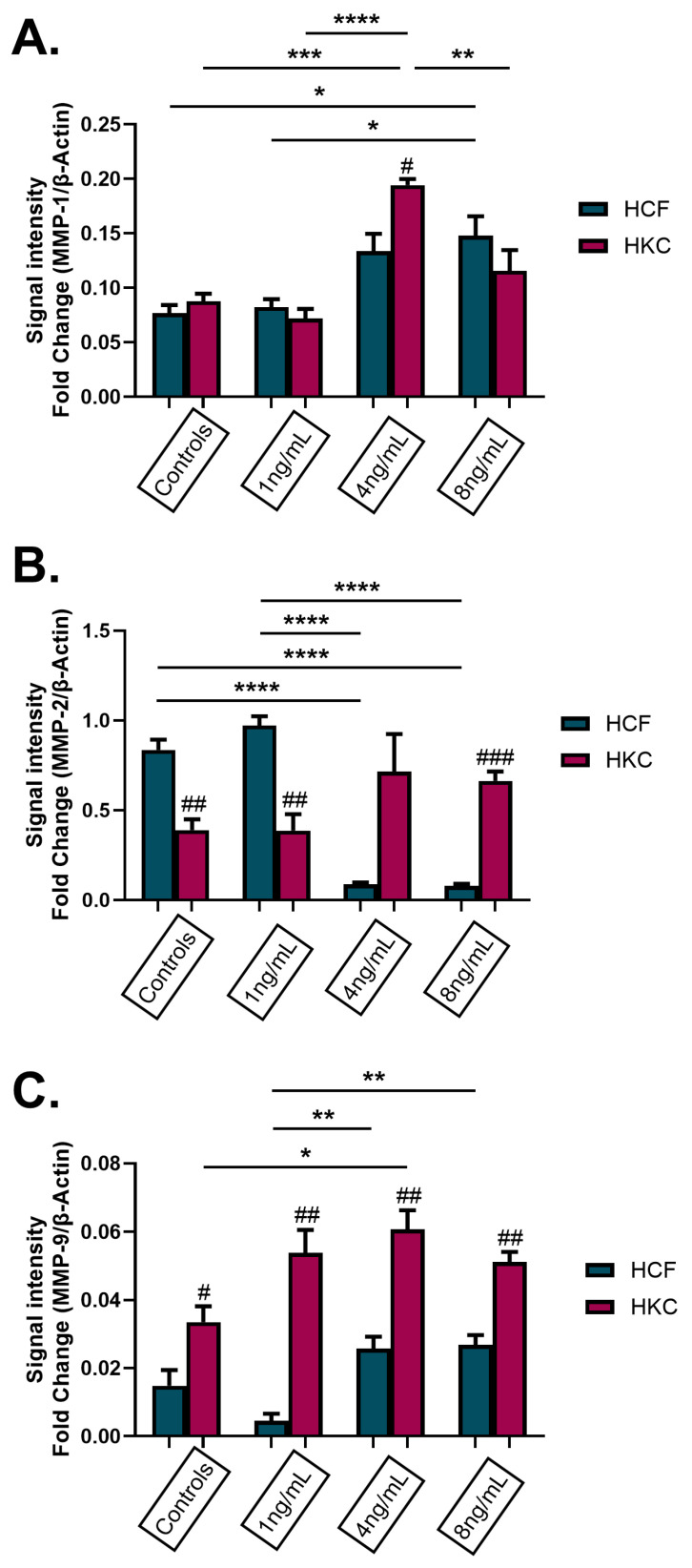
Protein expression of matrix metalloproteinases (MMPs) in 3D HCFs and HKCs after being stimulated with 1 ng/mL, 4 ng/mL, and 8 ng/mL GnRH for 4 weeks. Protein expression of (**A**) MMP-1, (**B**) MMP-2, and (**C**) MMP-9 in HCFs and HKCs stimulated with GnRH. Statistical significance based on a one-way ANOVA with Šídák’s multiple comparisons test. Post hoc significance indicated as * *p* < 0.05, ** *p* < 0.01, *** *p* < 0.001, and **** *p* < 0.0001. Statistical significance between HCFs and HKCs within the dose determined by the unpaired *t*-test with Welch’s correction. Significance indicated as # *p* < 0.05, ## *p* < 0.01, and ### *p* < 0.001.

To determine if matrix metalloproteinase (MMP) levels were influenced by GnRH stimulation, MMP-1, -2, and -9 protein expression was evaluated in 3D HCFs and HKCs after being stimulated with 1 ng/mL, 4 ng/mL, and 8 ng/mL rGnRH for 4 weeks. HCFs showed a significant increase in expression of MMP-1 when stimulated with 8 ng/mL of rGnRH compared to the control HCFs and HCFs stimulated with 1 ng/mL of rGnRH (*p* = 0.0042, Figure 7A). HKCs stimulated with 4 ng/mL of rGnRH showed a significant increase in MMP-1 expression when compared to their respective controls, as well as HKCs stimulated with 1 ng/mL and 8 ng/mL of rGnRH (*p* < 0.0001). In addition, HKCs stimulated with 4 ng/mL of rGnRH showed a significant increase in the expression of MMP-1 when compared to HCFs stimulated with 4 ng/mL of rGnRH (*p* = 0.0265). MMP-2 expression was significantly increased in HCFs when compared to HKCs in control (*p* = 0.0018) and stimulated with 1 ng/mL of rGnRH (*p* = 0.0029, Figure 7B). Interestingly, MMP-2 expression increased in HKCs compared to HCFs with stimulation of 4 ng/mL and 8 ng/mL of rGnRH, though this effect was only significant at 8 ng/mL (*p* = 0.0009). In addition, HCFs stimulated with 4 ng/mL and 8 ng/mL of rGnRH had significantly decreased MMP-2 expression when compared to control HCFs and HCFs stimulated with 1 ng/mL of rGnRH (*p* < 0.0001, Figure 7B). Expression of MMP-9 was significantly upregulated in HKCs compared to HCFs in controls (*p* = 0.0297) and when stimulated with all GnRH concentrations (1 ng/mL *p* = 0.0034, 4 ng/mL *p* = 0.0029, 8 ng/mL *p* = 0.0010, Figure 7C). In addition, expression of MMP-9 was significantly higher in HKCs stimulated with 4 ng/mL of rGnRH compared to HKC controls (*p* = 0.0173). Finally, there was a significant increase in MMP-9 expression in HCFs stimulated with 4 ng/mL and 8 ng/mL compared to HCFs stimulated with 1 ng/mL of rGnRH (*p* = 0.0017, Figure 7C).

## 4. Discussion

Keratoconus is one of the most significant ocular disorders associated with disease progression during pregnancy. It has been estimated that pregnancies complicate the pathological condition in patients pre-diagnosed with KC. The mechanisms of KC have not yet to be identified; however, hormonal pathological changes have been reported. The role of GnRH associated with KC in the human cornea is unknown. However, the fact that KC typically manifests during puberty and that pre-existing ocular pathologies are modified by pregnancy makes GnRH a hormone of interest for KC’s onset, development, and progression. This study investigated the role of GnRH, in vitro and in vivo, to better understand possible implications in the pathophysiology of KC. The plasma results for GnRH concentration of KCs were significantly lower than control patients when analyzed based on age, sex, and KC severity. For salivary results, the overall GnRH concentrations in KCs were lower than in control patients when comparing all samples and patients of ages greater than or equal to 46 y/o. This study showed that the GnRH findings were consistent in both plasma and saliva, albeit with GnRH detected at far lower concentrations in saliva compared to plasma (pg/mL versus ng/mL). Even though saliva samples can be the least invasive way to detect GnRH concentration, the saliva amylase enzyme used for the breakdown of starches could increase sample degradation and can account for the lower GnRH concentration. Therefore, this should be considered when using saliva as a diagnostic tool. Comparisons of the different GnRH concentration ELISA assays for different sample types should be further studied. GnRH release is known to be pulsatile. GnRH pulses are released on average every 30–120 min, which could account for the large ranges in circulating GnRH observed in this study [54,55]. As plasma and saliva was only sampled once, and the time when sample collection occurred was not recorded, it is unclear if these pulses affected our results. However, these results show that systemic GnRH concentration can correlate with conditions, such as KC. We found systemic GnRH is a quantifying soluble hormone that can be detected in both plasma and saliva using high-throughput commercially available ELISA, which can aid physicians with understanding the changes associated with this hormone that can exert physiological influence on KC progression.

In vitro, the role of GnRH in KC was evaluated by utilizing different concentrations of rGnRH (ranging from 1–500 ng/mL) on established 2D and 3D models using HCFs and HKCs. Features that differentiate the 2D and 3D models used in our paper include differences in the media supplementation, stiffness of the cell substrate, spatial organization of cells in the matrix, and length of culture conditions. Our 3D stromal model consists of spatially distributed corneal fibroblasts in a stratified, self-assembled extracellular matrix composed by collagen types I and V, which more closely resembles the in vivo conditions [56,57]. Further studies evaluating expression of collagen types I, III, and V are needed to determine the influence of GnRH stimulation on ECM secretion and deposition. In a human lifespan, GnRH secretion is observed after birth; however, it lowers in children, rises during puberty, and fluctuates during pregnancy. Therefore, because GnRH levels continuously fluctuate throughout the lifetime, we decided to initially use a wide rGnRH concentration range to assess which concentration had the most significant effect on the GnRH receptor, GnRHR, in the corneal stromal microenvironment for both HCFs and HKCs. Previous studies have reported the expression of GnRH in the retina of rat and zebrafish [61,62]. Further studies evaluating GnRH protein expression in human corneal tissue is warranted to validate expression patterns and functional responses in intact human corneal stroma.

When evaluating cultures using immunofluorescence and Western blots for protein expression of gonadotropin receptors, fibrotic markers, and MMPs-1, -2, and -9, the results showed significant differences between control and KC cells stimulated with rGnRH. GnRHR protein expression results showed a significant increase in HKCs stimulated with 1 ng/mL in both in vitro analysis, Western blot, and immunofluorescences. Interestingly, GnRHR expression in immunofluorescence showed increased expression in HKCs when stimulated with 8 ng/mL; however, this expression was decreased when the analysis was performed with Western blotting. The results show that it is always important to know what type of assay is being used and what each detects. Western blotting techniques detect proteins of interest extracted from cells by determining their molecular weights, resulting in an estimated quantity. Immunofluorescence technique detects the locations of proteins of interest in cell sections using fluorescent dye; however, estimated quantification is highly affected by the imaging system and staining quality. Therefore, Western blot results analyze the entire protein collected from the cells, making this technique more sensitive than immunofluorescence. In addition, gonadotropin receptor protein expression (LHR and FSHR) showed inverse results between HCFs and HKCs when stimulated with GnRH. Increasing levels of GnRH stimulate the production of LH and FSH, which results in effects on reproductive organs essential for maturation and fertility. The decrease in GnRH, except for during in vitro fertilization, which results in a GnRH agonist effect, produces a surge of FSH and LH to increase estrogen levels and facilitate ovulation. Even with the differences in the sensitivities of each assay, the effects of rGnRH stimulation on HKCs are evident in both methods. Further validation of GnRH levels in a larger secondary cohort is needed to support its use as a potential risk biomarker of KC. Indeed, representation of certain subgroups (e.g., KC females) was limited in the current study and should be expanded for future studies. Future studies should also focus on using GnRH as a therapeutic in the KC stromal microenvironment. For future studies, it would be interesting to look at GnRH levels in tears to identify if there is any direct contact between the hormone and its associated receptor and their responses in the corneal microenvironment of control and KC patients. Moreover, evaluating the abundance of GnRH in tears isolated from KC patients would provide additional support that differential systemic hormone levels reach the ocular surface. Exploration of other pregnancy-related hormones, including relaxin-2 and oxytocin [58,59,63], in KC subjects may provide insight regarding how dynamic fluctuations may influence corneal thickness.

Poor collagen fibers and proteoglycan remodeling are essential in increasing degenerative and irreversible changes in KC. Thus, it has been proposed as a significant reason for subsequent MMPs. MMPs are a family of enzymes implicated in normal tissue remodeling, wound healing, tumor invasion, and inflammatory processes [64,65]. In this study, MMP-1,-2, and -9 protein expression were significantly affected when simulated with rGnRH for both HCFs and HKCs. MMPs are secreted by a wide variety of epithelial and mesenchymal cell types. MMP-1’s role focuses on fibrous plaque disruption by degradation of interstitial collagens and thinning of the fibrous cap. MMP-2 can cleave gelatine and types I and IV of collagen, while MMP-9 cannot directly engage in the proteolysis of collagen I and digests collagen type IV [66,67,68]. MMP-9 is a marker of mobilization of tissue-bound growth factors and cytokines [65]. Impaired expression of matrix metalloproteinases has been associated with cytotrophoblast invasion and incomplete remodeling, demonstrated by placental ischemia as the main contributing factor in preeclampsia during pregnancy [69]. Pregnancy’s broad-spectrum association of pathological and physiological changes in the ocular system, including the risk factor for the progression of KC, highlights altered MMPs as a possible primary reason leading to collagen fibers and proteoglycans dysfunction in this study. Environmental factors, such as continued chronic eye rubbing [18,19,20], may be responsible for the disease development and progression of KC in correlation to GnRH dysfunction effects on tissue remodeling and wound healing; however, further investigation is needed.

## 5. Conclusions

In conclusion, GnRH receptors are widespread within the human body, including in ocular tissues. In addition, GnRH are multifaceted neuromodulators that not only play a pivotal role in reproduction but may also play a role in tissue remodeling and healing functions. Their roles within the human cornea should be further investigated; however, this study showed an association between GnRH and KC. Additional study of GnRH systems is likely to advance therapeutic options in other human ocular disorders, such as KC.

## Figures and Tables

**Table 1:** Western blot antibodies used in 2D and 3D cell culture. Adapted with permission from Escandon et al., 2023 [40]. American Society for Investigative Pathology.

Name	Source	Reactive	Dilution	Study	Antibody
**LHR**	ab125214 (Abcam, Cambridge, MA, USA)	Rabbit polyclonal	1:500	3D cell culture	Primary
**FSHR**	ab75200 (Abcam, Cambridge, MA, USA)	Rabbit polyclonal	1:500	3D cell culture	Primary
**GnRHR**	ab183079 (Abcam, Cambridge, MA, USA)	Rabbit polyclonal	1:500	2D + 3D cell culture	Primary
**MMP-1**	ab38929 (Abcam, Cambridge, MA, USA)	Rabbit polyclonal	1:500	3D cell culture	Primary
**MMP-2**	ab181286 (Abcam, Cambridge, MA, USA)	Rabbit monoclonal	1:500	3D cell culture	Primary
**MMP-9**	ab38898 (Abcam, Cambridge, MA, USA)	Rabbit polyclonal	1:500	3D cell culture	Primary
**α-SMA**	ab5694 (Abcam, Cambridge, MA, USA)	Rabbit polyclonal	1:500	3D cell culture	Primary
**EDA-Fn**	SAB4200784 (Millipore Sigma, Burlington, MA, USA)	Mouse monoclonal	1:700	3D cell culture	Primary
**β-actin**	ab184092 (Abcam, Cambridge, MA, USA)	Mouse monoclonal	1:2000	2D + 3D cell culture	Primary
**AlexaFluor 568**	ab175470 (Abcam, Cambridge, MA, USA)	Anti-rabbit IgG	1:2000	2D + 3D cell culture	Secondary
**AlexaFluor 647**	ab150107 (Abcam, Cambridge, MA, USA)	Anti-mouse IgG	1:2000	3D cell culture	Secondary

## Data Availability

Data supporting the findings are included within the paper and Supplemental Materials and are available from the corresponding author upon reasonable request.

## Supplementary Materials

The following supporting information can be downloaded at: https://www.mdpi.com/article/10.3390/cells13201704/s1. Figure S1: GnRH concentration in plasma for KC patients analyzed by KC treatment, including overall control results; Figure S2: GnRH concentration in plasma for KC patients analyzed by Pre and Post CXL treatment, including overall control results; Figure S3: GnRH analysis of saliva from control and KC patients with both saliva and plasma samples; Figure S4: GnRH concentration in saliva for KC patients analyzed by KC treatment, including overall control results; Figure S5: Protein expression of Gonadotropin-Releasing Hormone Receptor (GnRHR) in 2D HCF after being stimulated with 1 ng/mL, 2 ng/mL, 4 ng/mL, 6 ng/mL, 8 ng/mL, 10 ng/mL, 25 ng/mL, 50 ng/mL, 100 ng/mL and 500 ng/mL GnRH for 48 h.
