# Supplementary material for: Decreased Circulating Gonadotropin-Releasing Hormone Associated with Keratoconus
Source: Cells. 2024 Oct 15;13(20):1704. doi: 10.3390/cells13201704 (PMC11506063; doi:10.3390/cells13201704)
Supplement: Supplementary file 1 [file cells-13-01704-s001.zip › cells-3220405-supplementary.pdf]

Supplemental Materials:

Decreased Circulating Gonadotropin-Releasing Hormone Associated with Keratoconus

Paulina Escandon [1,2], Alexander J. Choi [1,2], Steve Mabry[1,2], Sarah E. Nicholas [1,2], Rebecca L. Cunningham[2], David A. Murphy[4], Liam Redden[4], Kamran M. Riaz [4], Tina B. McKay [5,*], and Dimitrios Karamichos [1,2,3*]

Supplementary Figure S1. GnRH concentration in plasma for KC patients analyzed by KC treatment, including overall control results.

Supplementary Figure S2. GnRH concentration in plasma for KC patients analyzed by Pre and Post CXL treatment, including overall control results.

Supplementary Figure S3. GnRH analysis of saliva from control and KC patients with both saliva and plasma samples.

Supplementary Figure S4. GnRH concentration in saliva for KC patients analyzed by KC treatment, including overall control results.

Supplementary Figure S5. Protein expression of Gonadotropin-Releasing Hormone Receptor (GnRHR) in 2D HCF after being stimulated with 1 ng/mL, 2ng/mL, 4 ng/mL, 6 ng/mL, 8 ng/mL, 10 ng/mL, 25 ng/mL, 50 ng/mL, 100 ng/mL and 500 ng/mL GnRH for 48 hours.

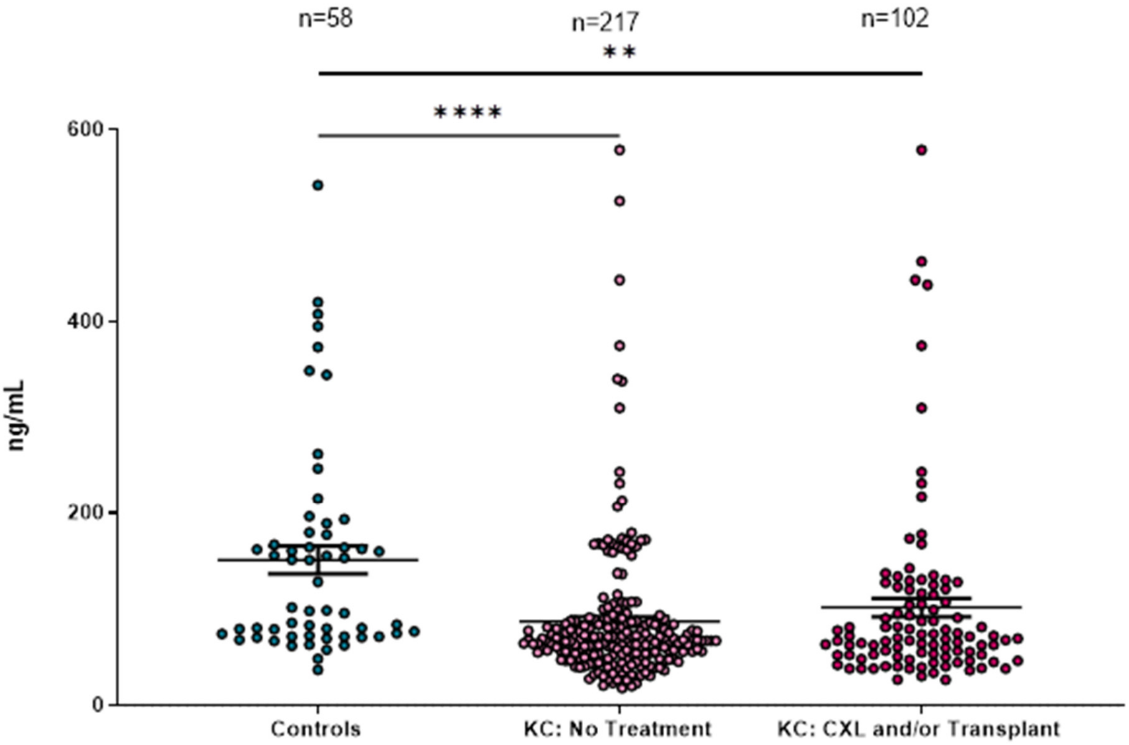

Supplementary Figure S1. GnRH concentration in plasma for KC patients analyzed by KC treatment, including overall control results. **p<0.01, and ****p<0.0001.

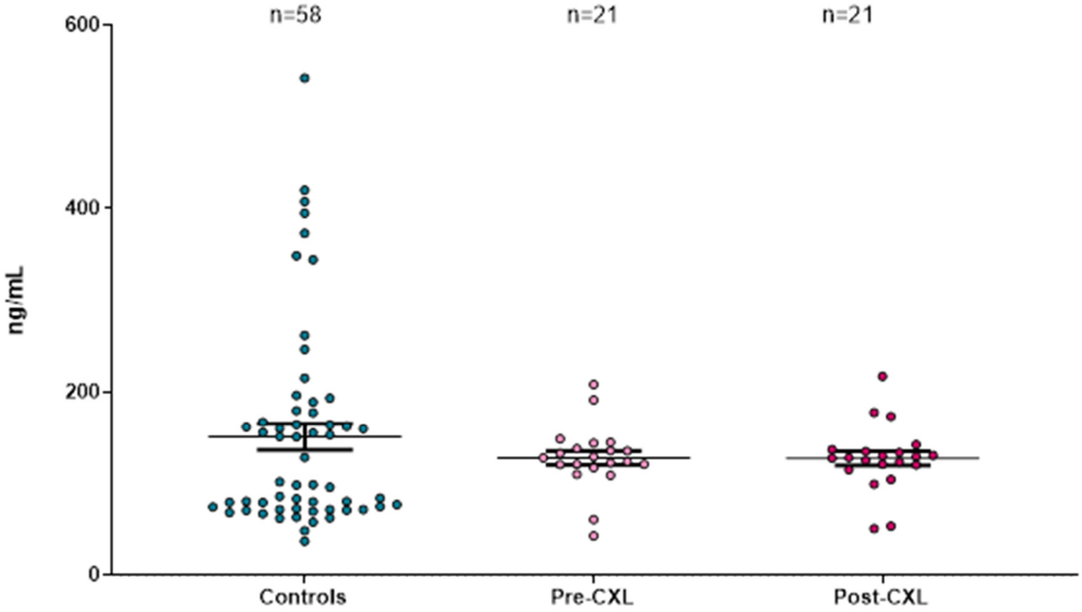

Supplementary Figure S2. GnRH concentration in plasma for KC patients analyzed by Pre and Post CXL treatment, including overall control results.

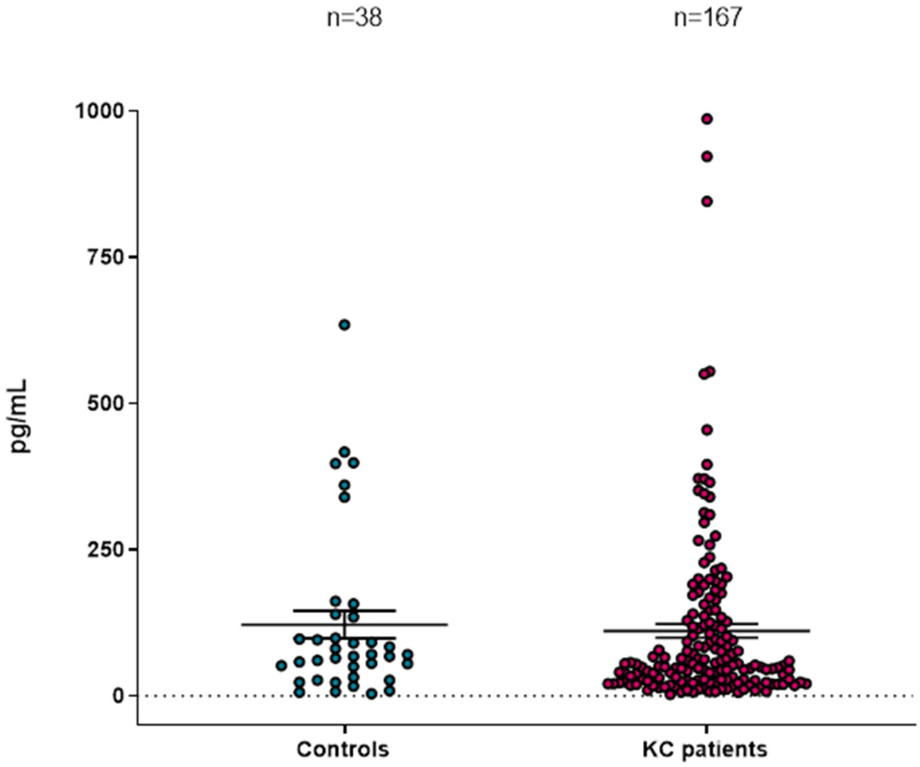

Supplementary Figure S3. GnRH analysis of saliva from control and KC patients with both saliva and plasma samples.

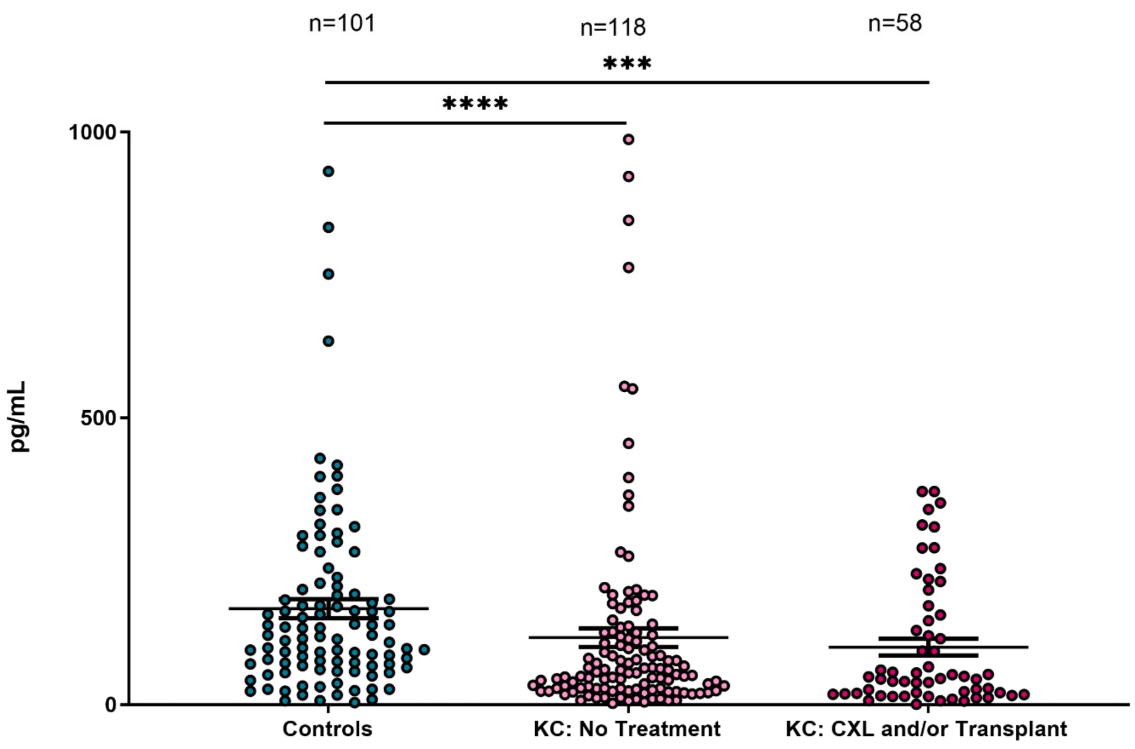

Supplementary Figure S4. GnRH concentration in saliva for KC patients analyzed by KC treatment, including overall control results. ***p<0.001, and ****p<0.0001.

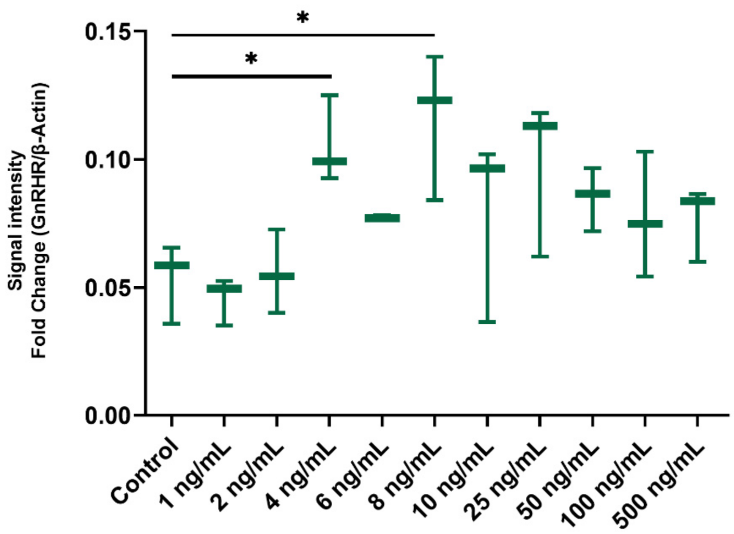

Supplementary Figure S5. Protein expression of Gonadotropin-Releasing Hormone Receptor (GnRHR) in 2D HCF after being stimulated with 1 ng/mL, 2ng/mL, 4 ng/mL, 6 ng/mL, 8 ng/mL, 10 ng/mL, 25 ng/mL, 50 ng/mL, 100 ng/mL and 500 ng/mL GnRH for 48 hours. *p<0.05.
